# Supplementary material for: Differential regulation by CD47 and thrombospondin-1 of extramedullary erythropoiesis in mouse spleen
Source: eLife. 2024 Jul 9;12:RP92679. doi: 10.7554/eLife.92679 (PMC11233134; doi:10.7554/eLife.92679)
Supplement: Figure 4—source data 1. [file elife-92679-fig4-data1.docx]

**Figure 4-source data 1. Differential expression of erythropoietic, stem cell, and proliferation associated markers in cell clusters 12 and 14.** Percentages of cells with detectable mRNA expression are presented for the indicated erythropoietic, stem and proliferative markers in cluster 12 and cluster 14 in WT, Cd47*^−/−^*, and *Thbs1^−/−^* spleens.

| **Cluster** | **Gene** | **% positive *Cd47*^-/-^ cells** | **% positive *Thbs1*^-/-^ cells** | **% positive WT cells** |
| --- | --- | --- | --- | --- |
| **12** | *Klf1* | 88.0 | 79.7 | 89.1 |
| **14** | *Klf1* | - | - | - |
| **12** | *Aqp1* | 80.3 | 78.1 | 80.4 |
| **14** | *Aqp1* | - | - | - |
| **12** | *Tfrc* | 44 | 34.4 | 35.9 |
| **14** | *Tfrc* | 13.5 | 10.7 | 9.1 |
| **12** | *Epor* | 58.5 | 51.6 | 65.2 |
| **12** | *Ermap* | 66.9 | 65.6 | 64.1 |
| **12** | *Gata1* | 77.1 | 53.1 | 73.9 |
| **12** | *Mki67* | 74.6 | 64.1 | 57.6 |
| **14** | *Mki67* | 41.1 | 9.3 | 24.2 |
| **12** | *Kit* | 80.6 | 75.0 | 67.4 |
| **14** | *Kit* | 30.7 | 20.0 | 13.6 |
| **12** | *Xpo1* | 67.6 | 53.1 | 38.0 |
| **14** | *Xpo1* | 38.0 | 30.7 | 25.8 |
| **12** | *Ranbp1* | 95.8 | 98.4 | 91.3 |
| **14** | *Ranbp1* | 71.2 | 54.7 | 69.7 |
| **12** | *Ranbp2* | 85.9 | 64.1 | 47.8 |
| **14** | *Ranbp2* | 57.7 | 38.7 | 34.8 |
| **12** | *Nr3c1* | 63.4 | 68.8 | 42.4 |
| **14** | *Nr3c1* | 47.2 | 64.0 | 28.8 |
| **12** | *Ddx46* | 78.9 | 87.5 | 50.0 |
| **14** | *Ddx46* | 50.9 | 50.7 | 25.8 |
