# Supplementary material for: Differential regulation by CD47 and thrombospondin-1 of extramedullary erythropoiesis in mouse spleen
Source: eLife. 2024 Jul 9;12:RP92679. doi: 10.7554/eLife.92679 (PMC11233134; doi:10.7554/eLife.92679)
Supplement: Figure 4—source data 2. [file elife-92679-fig4-data2.docx]

**Figure 4-source data 2. Differential mRNA expression of the nuclear export protein Xpo1 and nuclear pore protein synthesis instructor Ranbp2, which increase erythropoiesis by stabilizing Gata1 in the nucleus, in cluster12 and CD34^+^ and CD34^−^ subsets of cluster 12 cells.**

| **Cell cluster** | **mRNA** | ***Cd47*^−/−^ vs WT** | | ***Thbs1*^−/−^ vs WT** | |
| --- | --- | --- | --- | --- | --- |
|  |  | **p-value** | **Avg FC** | **p-value** | **Avg FC** |
| C12 | Xpo1 | 3.3x10^-8^ | 1.39 | 0.0069 | 1.25 |
| C12 (CD34^−^) | Xpo1 | 2.3x10^-8^ | 1.42 | 0.010 | 1.25 |
| C12 (CD34^+^) | Xpo1 | NS |  | -* |  |
| C14 | Xpo1 | NS |  | NS |  |
| C12 | Ranbp2 | 4.3x10^-14^ | 1.69 | NS |  |
| C12 (CD34^−^) | Ranbp2 | 3.8x10^-15^ | 1.76 | NS |  |
| C12 (CD34^+^) | Ranbp2 | NS |  | -* |  |
| C14 | Ranbp2 | 0.0055 | 1.29 | NS |  |

*Insufficient CD34^+^ *Thbs1^−/−^* cells to calculate
