# Supplementary material for: Differential regulation by CD47 and thrombospondin-1 of extramedullary erythropoiesis in mouse spleen
Source: eLife. 2024 Jul 9;12:RP92679. doi: 10.7554/eLife.92679 (PMC11233134; doi:10.7554/eLife.92679)
Supplement: Figure 7—source data 1. [file elife-92679-fig7-data1.docx]

**Figure 7−source data 1. Differential expression of erythropoietic, stem cell, and proliferation associated markers in reclustered erythroid and T cell clusters.** The percent of cells positive for expression of the indicated genes in WT, *Cd47^−/−^*, and *Thbs1^−/−^* cells is presented in the indicated clusters. (-) indicates that no cells in the cluster expressed detectable levels of the indicated gene.

| **Cluster** | **Gene** | **% positive *Cd47*^−/−^ cells** | **% positive *Thbs1*^−/−^ cells** | **% positive WT cells** |
| --- | --- | --- | --- | --- |
| **Erythroid** | *Klf1* | 91.5 | 84.5 | 99.1 |
| **T cells** | *Klf1* | 25.6 | 21.4 | 26.0 |
| **Erythroid** | *Aqp1* | 78.9 | 83.0 | 84.5 |
| **T cells** | *Aqp1* | 32.2 | 21.4 | 28.9 |
| **Erythroid** | *Tfrc* | 45.8 | 37.9 | 36.7 |
| **T cells** | *Tfrc* | - | - | - |
| **Erythroid** | *Epor* | 60.1 | 56.9 | 64.4 |
| **Erythroid** | *Ermap* | 70.7 | 65.6 | 64.4 |
| **T cells** | *Ermap* | 26.4 | 23.9 | 20.2 |
| **Erythroid** | *Gata1* | 80.8 | 58.6 | 75.6 |
| **Erythroid** | *Mki67* | 77.1 | 69.0 | 58.9 |
| **T cells** | *Mki67* | - | - | - |
| **Erythroid** | *Kit* | 80.1 | 77.6 | 68.9 |
| **T cells** | *Kit* | - | - | - |
| **Erythroid** | *Xpo1* | 69.4 | 56.9 | 37.8 |
| **T cells** | *Xpo1* | 21.7 | 23.9 | 16.8 |
| **Erythroid** | *Ranbp1* | 96.7 | 98.4 | 91.1 |
| **T cells** | *Ranbp1* | 54.3 | 61.5 | 60.1 |
| **Erythroid** | *Ranbp2* | 86.7 | 67.2 | 48.9 |
| **T cells** | *Ranbp2* | 46.1 | 36.8 | 27.7 |
| **Erythroid** | *Nr3c1* | 63.5 | 67.2 | 41.1 |
| **T cells** | *Nr3c1* | 44.2 | 47.9 | 27.7 |
| **Erythroid** | *Ddx46* | 81.5 | 86.2 | 50.0 |
| **T cells** | *Ddx46* | 55.0 | 59.8 | 33.5 |
| **Erythroid** | *Hba-a1* | 23.6 | 6.9 | 25.6 |
| **T cells** | *Hba-a1* | - | - | - |
